# Loss of ISWI Function in *Drosophila* Nuclear Bodies Drives Cytoplasmic Redistribution of *Drosophila* TDP-43

**DOI:** 10.3390/ijms19041082

**Published:** 2018-04-04

**Authors:** Luca Lo Piccolo, Rosa Bonaccorso, Andrea Attardi, Lorenzo Li Greci, Giulia Romano, Martina Sollazzo, Giorgio Giurato, Antonia Maria Rita Ingrassia, Fabian Feiguin, Davide F. V. Corona, Maria Cristina Onorati

**Affiliations:** 1 STEBICEF, Viale delle Scienze, Edificio 16, Università degli Studi di Palermo, 90128 Palermo, Italy; lucalopiccolo@gmail.com (L.L.P.); rosabonaccorsog@gmail.com (R.B.); andrea-attardi@libero.it (A.A.); lorenzoligreci@gmail.com (L.L.G.); martina.sollazzo94@gmail.com (M.S.); aingrassia@dti.telethon.it (A.M.R.I.); davide.corona@unipa.it (D.F.V.C.); 2 International Centre for Genetic Engineering and Biotechnology Padriciano 99, 34149 Trieste, Italy; Giulia.Romano@icgeb.org (G.R.); fabian.feiguin@icgeb.org (F.F.); 3 Genomix4Life srl, Department of Medicine, Surgery and Dentistry “Scuola Medica Salernitana”, University of Salerno, 84081 Baronissi (SA), Italy; ggiurato@unisa.it

**Keywords:** *Drosophila*, nuclear body, omega speckles, dTDP-43, hnRNPs, omega speckles, neurodegenerative diseases, gene expression, gene regulation

## Abstract

Over the past decade, evidence has identified a link between protein aggregation, RNA biology, and a subset of degenerative diseases. An important feature of these disorders is the cytoplasmic or nuclear aggregation of RNA-binding proteins (RBPs). Redistribution of RBPs, such as the human TAR DNA-binding 43 protein (TDP-43) from the nucleus to cytoplasmic inclusions is a pathological feature of several diseases. Indeed, sporadic and familial forms of amyotrophic lateral sclerosis (ALS) and fronto-temporal lobar degeneration share as hallmarks ubiquitin-positive inclusions. Recently, the wide spectrum of neurodegenerative diseases characterized by RBPs functions’ alteration and loss was collectively named proteinopathies. Here, we show that TBPH (TAR DNA-binding protein-43 homolog), the *Drosophila* ortholog of human TDP-43 TAR DNA-binding protein-43, interacts with the arcRNA hsrω and with hsrω-associated hnRNPs. Additionally, we found that the loss of the omega speckles remodeler ISWI (Imitation SWI) changes the TBPH sub-cellular localization to drive a TBPH cytoplasmic accumulation. Our results, hence, identify TBPH as a new component of omega speckles and highlight a role of chromatin remodelers in hnRNPs nuclear compartmentalization.

## 1. Introduction

In the past year, several studies have indicated that heterogeneous ribonucleoprotein (hnRNPs) are linked to neurodegenerative diseases such as spinal muscular atrophy (SMA), amyotrophic lateral sclerosis (ALS), Alzheimer’s disease (AD), and fronto-temporal lobar degeneration (FTLD). These conditions share the presence of cytoplasmic or nuclear aggregates of RNA-binding proteins (RBPs) and RNA [1,2,3,4,5,6,7,8]. For this feature, they are referred to as *proteinopathies*. One of the proteins most involved in neurodegenerative diseases is the TAR DNA-binding protein 43 (TDP-43). Classified as the major disease protein in cytoplasmic inclusions [1,2,4], human TDP-43 is an hnRNP [8,9,10] ubiquitously expressed in all tissues but highly expressed in the brain and kidney [11].

In large motor neurons, TDP-43 plays an important role in mRNA transport as a neuronal activity responsive factor in dendrites [12]. TDP-43 regulates synaptic plasticity by governing the transport and splicing of synaptic mRNAs [13] and it is also involved in different important functions that regulate RNA metabolism as well as transcriptional repression, exon skipping, RNA splicing and transport/stability, microRNA expression, and RNA translation [14,15,16,17].

Mutations in both TDP-43 N- and the C-terminus are associated with the formation of large ubiquitin-positive TDP-43 inclusions in the brains of patients affected by ALS-FTLD [18,19]. TDP-43-positive inclusions are also detected in neurodegenerative pathological conditions in the absence of mutations, raising the possibility that other factors such as post-translational modifications might be involved in the altered nucleo-cytoplasmic shuttling of TDP-43 [20]. 

*Drosophila melanogaster* is fruitfully used as a model for neurodegenerative diseases, especially for those caused by aberrant hnRNPs activities. *Drosophila* encodes at least 14 major hnRNPs, with structural and functional orthologs in mammals, most of them associated in the omega speckles, as well as Hrb87F and Squid, the orthologs of the human hnRNPA2/B1 and hnRNPA1. 

Omega speckles belong to the group of large granular structures known as nuclear bodies (NBs) [21] which have roles in biogenesis, maturation, storage and the sequestration of specific RNAs, proteins, and ribonucleoprotein complexes [8,22]. The structural organization of omega speckles is guaranteed by the long architectural non-coding RNA (arcRNA) heat-shock response omega (hsrω) [23,24,25], which operates as an essential scaffold or platform of nuclear bodies such as the long non-coding RNA NEAT1 for paraspeckles [26] and Sat III for nuclear stress bodies (nSBs) [27]. 

In a previous publication, a genetic and functional interaction between hsrω and the chromatin remodeler ISWI was observed [28]. ISWI is the catalytic subunit of several ATP-dependent chromatin-remodeling complexes, is highly conserved during evolution, and is essential for cell viability [29,30]. ISWI is essential for the correct organization of the omega speckles, because loss of ISWI function can lead to severe changes in the structure of nuclear bodies and misregulation of RNA processing [28,31].

In *Drosophila*, TBPH, the ortholog of the human TDP-43 [11] plays a role in neuronal and neuromuscular development in different fly-model systems [1,2,4,8,32]. Moreover, hnRNPA2/B1 and hnRNPA1 interact with TDP-43 to regulate RNA metabolism [9] HnRNPA2B1 and to engage into stress granules (SGs) in the context of multi-system proteinopathy [6].

In this study, we examined the interaction of TBPH with some omega speckle-associated hnRNPs and the arcRNA hsrω. Moreover, we analyzed the role of ISWI in the sub-cellular localization of TBPH. We demonstrate that TBPH physically interacts with Squid and Hrb87F hnRNPs and with arcRNA hsrω. Interestingly, we found that ISWI nullisomy can alter the TBPH sub-cellular localization to promote its cytoplasmic build-up. Similar to other hsrω-associated hnRNPs, we herein show that the ISWI function is critical for the TBPH engagement in hsrω and plays a crucial role in hnRNP nucleo-cytoplasmic shuttling. These results might open a new scenario in the study of neurodegenerative diseases caused by abnormal hnRNP cytoplasmic accumulation, especially in those sporadic familial cases of neurodiseases in which TDP-43 mutations are absent. 

## 2. Results and Discussion

### 2.1. hnRNP TBPH Interacts with Squid and Hrb87F hnRNPs In Vivo

**Figure 1:**
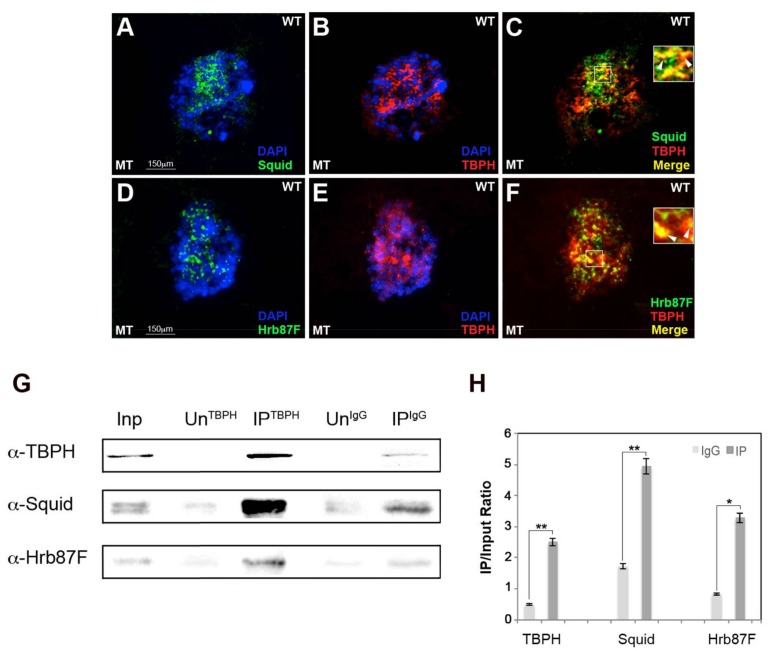
TBPH interacts with Squid and Hrb87F hnRNPs in vivo (**A**–**F**), confocal images of double immunofluorescence showing that in Malpighian tubules (MT) cells TBPH (red) co-localizes with Squid hnRNP (green) (**A**–**C**), and arrowheads in higher magnification white box in (**C**) and with Hrb87F (green) (**D**–**F**), and arrowheads in higher magnification white box in (**F**). Single nuclei are shown in detail as representative of several observations across three biological replicates, (**G**) Co-immunoprecipitation assay was conducted on brain larval nuclear extracts using an affinity purified TBPH antibody (see Material and Methods). The immunoprecipitated material was analyzed by Western blotting using anti-TBPH, Squid, Hrb87F, and GAPDH (Glyceraldeyde phosphate dehydrogenase) antibodies. Generic rabbit IgG was used as control. Inp = Input, Un^TBPH^ = Unbound IP^TBPH^ material, Un^IgG^ = Unbound IP^IgG^ material, IP = Immunoprecipitated material. (**H**) Quantification of the signals intensity. The intensity of Co-immunoprecipitation signals was quantified using ImageJ. The experiment was performed considering five biological replicates. The error bars show the standard deviation. Unpaired Student’s *t*-test was performed to assay statistical significance; * 0.01 ≤ *p*-value ≤ 0.05; ** *p*-value < 0.01.

The human orthologs of Squid and Hrb87F proteins interact with TDP-43 to function cooperatively in RNA metabolism regulation [14]. We addressed whether TBPH interacts with Hrb87F and Squid in *Drosophila* cells as well. We conducted double-immunofluorescence for TBPH/Hrb87F and TBPH/Squid and, as shown in Figure 1, we found that TBPH co-localizes in Malpighian tubules (MT) with Squid (Figure 1A–C) and Hrb87F hnRNPs in vivo ( Figure 1D–F and Figure S1). 

To confirm these interactions, we conducted a co-immunoprecipitation assay using anti-TBPH antibody starting from wild-type (WT) fresh larval nuclear extracts [33]. As shown in Figure 1G,H, Squid and Hrb87F are enriched in TBPH pulled-down fractions. 

Several in vitro experiments through proteomic studies [14,34] and co-immunoprecipitation assay in HEK293 cells [35] showed that in human cells TDP-43 interacts with the *Drosophila* orthologs of Squid and Hrb87F hnRNPs. Here, we confirmed these results in vivo, showing that in *Drosophila* tissue TBPH also interacts with Squid and Hrb87F hnRNPs.

### 2.2. TBPH Physically and Functionally Interacts with the hsrω arcRNA In Vivo and In Vitro

**Figure 2:**
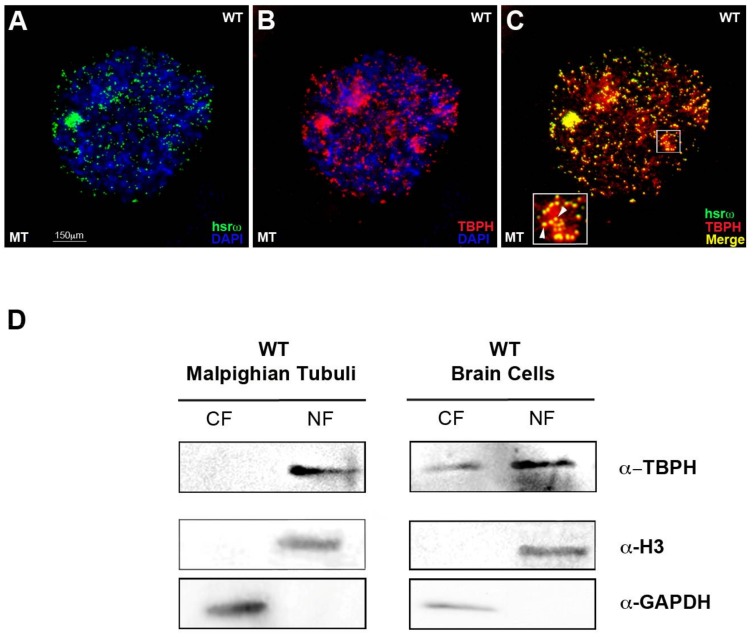
Immuno-FRISH showing that TBPH physically interacts with the hsrω arcRNA in vivo. (**A**–**C**) Immunostaining for TBPH (red) combined with FRISH for hsrω RNA (green) on wild-type (WT) in MT partial squashed nuclei. Arrowheads in higher magnification white box in (**C**) show co-localization points between TBPH protein and the arcRNA (single nuclei are shown in detail as representative of several observations across 3 biological replicates). (**D**) TBPH protein distribution between the nucleus and cytoplasm is different in WT tissues: in MT tissues (left panel) TBPH is in the nucleus (NF) while in BCs cells (right panel) the protein is present both in cytoplasm (CF) as well as in the nucleus (NF). Anti-GAPDH and anti-H3 were used as internal control to normalize CF and NF.

Considering the co-localization between TBPH and Squid/Hrb87F hnRNPs, we wondered whether TBPH and hsrω could physically interact as well. To answer this question, we conducted an immunofluorescence and fluorescence RNA in situ hybridization (Immuno-RNA–FISH or Immuno-FRISH). We observed a physical interaction between TBPH and the arcRNA hsrω in MT in vivo (Figure 2A–C).

As hnRNPs are known to shuttle between nucleus and cytoplasm, we performed Western blot analysis using a previously described method to produce in a single experiment nuclear and cytoplasmic protein fractions (NF and CF) [33]. These experiments were performed using MT and brain cells (BCs). Omega speckles are present in all the larval and adult *Drosophila* cell-type tissues but we used cells from Malpighian tubules for their large nuclear size, which allow a better understanding of nuclear bodies’ distribution, as well as the eventual hsrω-interacting protein subcellular localization. We also characterized BCs, as TBPH is largely expressed and has fundamental roles in the brain.

The localization of TBPH in BCs is predominantly localized in the nucleus (Figure 2D, left panel), but in BCs there is also a fraction of TBPH protein in the cytoplasm (Figure 2D, right panel).

**Figure 3:**
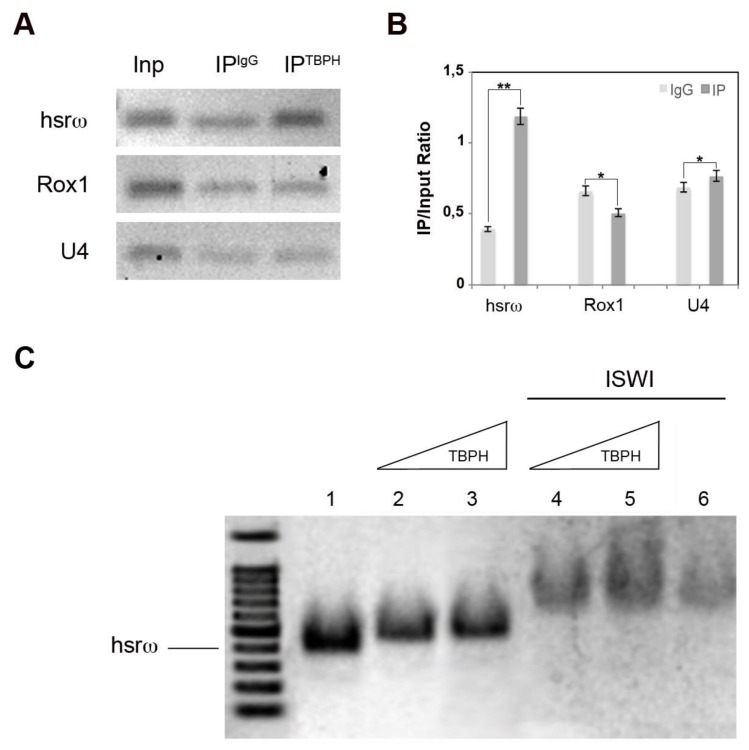
TBPH physically and functionally interacts with the hsrω ncRNA in vitro. (**A**) Cross-linking RNA-immuno-precipitation (CLIP-RIP) assay was carried out with affinity purified anti-TBPH antibody on fixed larval nuclear extracts from the brain. The immunoprecipitate was analyzed by real-time polymerase chain reaction (RT-PCR) using primers for the 280 bp tandem repeat unit of the nuclear hsrω ncRNA; primers amplifying the U4 and Rox1 ncRNAs were used as specificity controls. Inp = input, IP^TBPH^ = immunoprecipitated material from anti-TBPH CLIP, IP^IgG^ = immunoprecipitated material from IgG CLIP; (**B**) The intensity of PCR signals was quantified using ImageJ. The experiment was performed considering five biological replicates. The error bars show the standard deviation. Unpaired Student’s *t*-test was performed to assay statistical significance; * 0.01 ≤ *p*-value ≤ 0.05; ** *p*-value < 0.01; (**C**) TBPH hnRNP binds α*^32^*P labeled hsrω-n arcRNA in electrophoresis shift mobility assay (EMSA). ISWI modulates the TBPH-hsrω interaction, as shown by changes in the gel shift. Binding reactions were performed at different stoichiometric ratios for hsr-ω/TBPH: 1:2 (lanes 2 and 4), 1:50 (lanes 3 and 5) and constant ratio for hsr-ω/ISWI: 1:20 (lanes 4, 5, and 6). Lane 1 shows the α*^32^*P hsrω-n probe only.

To rule out that the physical association observed between TBPH and hsrω was due to fortuitous interactions occurring during nuclear extract preparation, we conducted a cross-linking RNA-immuno-precipitation (CLIP-RIP) biochemical assay [28] using the anti-TBPH antibody on fixed larval nuclear extracts from brain cells. The CLIP-RIP data confirmed the specific interaction between TBPH and hsrω in the nuclear extract from the brain cells (+3.05-fold), as observed in Figure 3A,B compared to Rox1 (+0.77-fold) and U4 (+1.1-fold), two other abundant nuclear non-coding RNAs.

We, thus, demonstrated that TBPH, as previously reported for Hrb87F and Squid [36], is able to bind the hsrω RNA in vitro. Furthermore, using a gel shift assay employing an hsrω-n repeat unit (280b) transcribed in vitro and a full-length recombinant TBPH, we show that TBPH effectively retards hsrω RNA gel mobility (Figure 3C lane 1–3). Finally, as seen for Hrb87F and Squid hnRNPs [36], the addition of ISWI protein in the reaction is a strong modulator of the interaction between TBPH-hsrω, changing the gel shift delay (Figure 3C lane 4–6).

In conclusion, our experiments confirmed the interaction of TBPH with hsrω arcRNA, Squid and Hrb87F hnRNPs in the omega speckles context. These results strongly suggest that, like Hrb87F and Squid [28], TBPH is another hnRNP belonging to the omega speckles complexes. Moreover, as shown for Squid and Hrb87F hnRNPs, ISWI function is essential for the modulation of TBPH/hsrω interaction.

### 2.3. Loss of ISWI Chromatin Remodeler Function Drives TBPH Cytoplasmic Redistribution in the Omega Speckles

The chromatin remodeler ISWI is essential for a correct organization of the nucleoplasmic omega speckles [28]. Indeed, the organization and distribution of omega speckles are profoundly altered in *ISWI* null mutants when compared to wild-type cells. Omega speckles lose their dot shape and assume a trail shape distribution in the nucleus [28], suggesting a severe defect in their maturation or organization. Squid and Hrb87F hnRNPs also form trail-like structures in the nucleus of *ISWI* null mutants, showing that not only the distribution of the hsrω arcRNA, but also that of omega speckle-associated hnRNPs is compromised [28].

**Figure 4:**
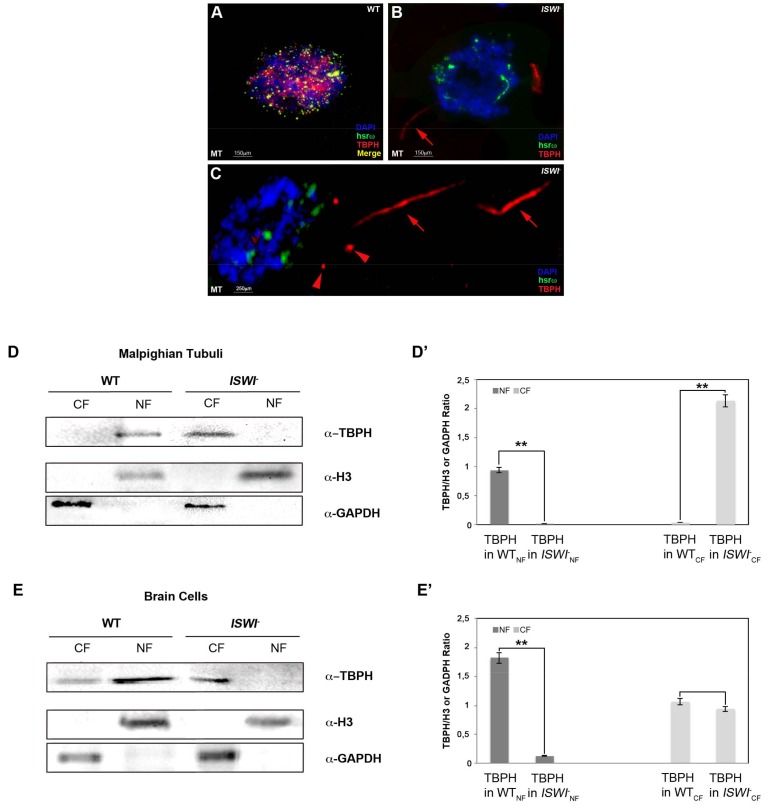
Loss of ISWI causes severe changes in TBPH localization. (**A**–**C**) Compared to WT (**A**), loss of ISWI dramatically changes TBPH protein (red) distribution between the nucleus and cytoplasm in MT of *ISWI^-^* null mutants (**B**), and higher magnification of other cells in (**C**). TBPH loses its co-localization with hsrω (green) and it is predominantly distributed in the cytoplasm (red arrows). Moreover, TBPH in the cytoplasm seems to be organized in different trail-like structure (arrows) or dot (arrowheads) (**C**), (**D**,**E**) Cellular fractionation experiments analyzed by Western blots confirming the data for MT cells (**D**), as well as BCs (**E**). Anti-GAPDH and anti-H3 were used as internal control to normalize CF and NF. (**D’**,**E’**) The intensity of Western blot signals was quantified using ImageJ. The experiment was performed considering five biological replicates. The error bars show the standard deviation. Unpaired Student’s *t*-test was performed to assay statistical significance; * 0.01 ≤ *p*-value ≤ 0.05; ** *p*-value < 0.01.

Therefore, we analyzed the distribution of TBPH protein in *ISWI* null mutants to check if loss of ISWI could influence TBPH organization in omega speckles NBs as for Hrb87F and Squid hnRNPs [28]. Remarkably, we found that compared to wild-type cells, loss of ISWI function changes TBPH distribution in the context of omega speckles, inducing a dramatic alteration of TBPH sub-cellular localization (Figure 4). While in WT MT TBPH immunoreactive spots are nucleus limited, in *ISWI* null mutants’ MT we detected cytoplasmic TBPH-positive spots and trails (Figure 4, compare A and B). Of note, these cytoplasmic spots show to be organized in different shapes, as indicated by arrows and arrowheads (Figure 4C).

We confirmed these data in vitro by Western blot of nuclear and cytoplasmic fractions in WT versus *ISWI* null mutant MT and BCs (Figure 4D,E). In detail, we show that in *ISWI* null mutant the TBPH protein in MT disappears from the nucleus while moving to the cytoplasm, where the TBPH abundance increased compared to the WT NF (+2.37-fold) (Figure 4D’). We also observed a similar phenomenon in BCs where we found that in *ISWI* null mutant TBPH disappears from the NF while its CF amount is not significantly changed compared to WT (−0.09-fold) (Figure 4E’).

**Figure 5:**
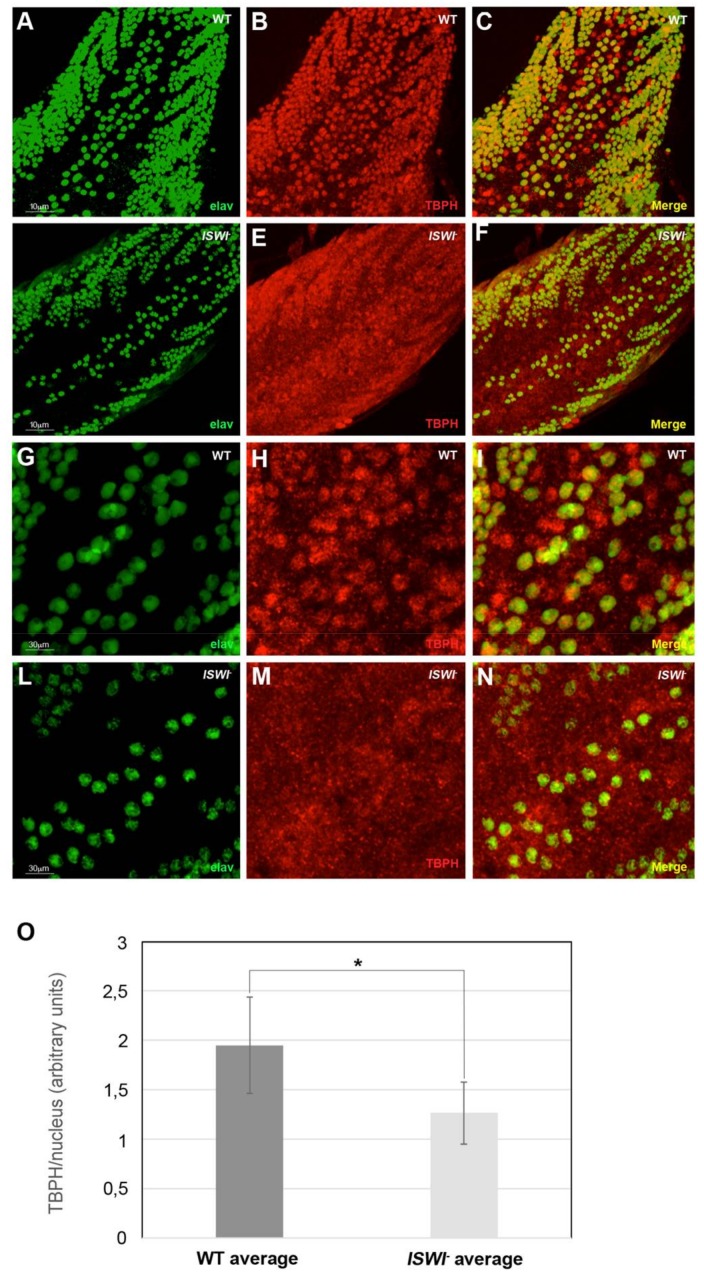
Changes in the distribution of TBPH hnRNP in *ISWI* null mutant compared to WT in the ventral ganglion of 3rd instar larvae. (**A**–**L**) Immunostaining on the ventral ganglia dorsal medial clusters of motoneuron nuclei in third instar wild-type (**A**–**C**,**G**–**I** magnification) and *ISWI^-^* null mutant larvae (**D**–**F**,**L**–**N** magnification). The mean intensity of TBPH (in red) in the motoneuron nuclei stained with neuronal nuclear protein anti-Elav (green) is significantly reduced in *ISWI^-^* null mutants (**F**,**N** magnification) compared to wild-type (**C**,**I** magnification), as visible also in the merge (**C**,**F**). (**O**) Quantification of the intensity of the immunofluorescence signals. The error bars show the standard deviation. Unpaired Student’s *t*-test was performed to assay statistical significance; * 0.01 ≤ *p*-value ≤ 0.05; ** *p*-value < 0.01.

Analyzing in detail the ventral ganglion of WT and *ISWI* null larvae (Figure 5A–F,G–N magnification), we observed that the mean intensity of TBPH in motoneuron nuclei of *ISWI* null mutants is reduced compared to WT (−1.52-fold) (Figure 5O).

### 2.4. Squid hnRNP Influences TBPH Distribution between the Nucleus and Cytoplasm

**Figure 6:**
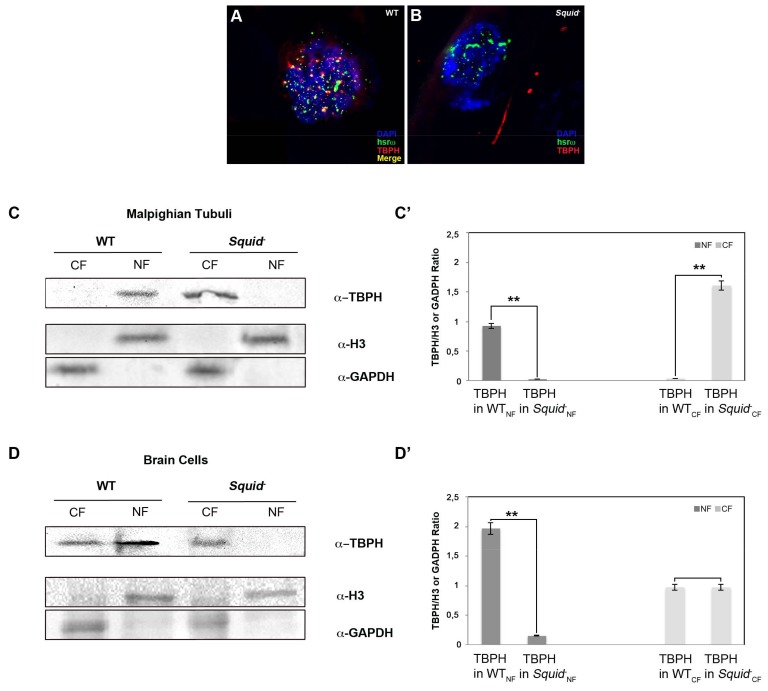
Loss of Squid causes severe changes in TBPH localization. (**A**,**B**) Compared to WT (**A**), loss of Squid changes TBPH protein (red) distribution between nucleus and cytoplasm in MT of *Squid^-^* null mutants (**B**,**C**). Indeed, TBPH loses its co-localization with hsrω (green) and it is predominantly distributed in the cytoplasm (Red arrows). (**C**,**D**) Western blots confirming the data for MT cells (**C**) as well as BCs (**D**). (**C’**,**D’**) The intensity of Western blot signals was quantified using ImageJ. The experiment was performed considering five biological replicates. The error bars show the standard deviation. Unpaired Student’s *t*-test was performed to assay statistical significance; * 0.01 ≤ *p*-value ≤ 0.05; ** *p*-value < 0.01.

Interestingly, loss of Squid in the *Squid^-^* null mutant also affects the cellular distribution of TBPH and causes its aberrant cytoplasmic localization in MT. While in WT MT TBPH immunoreactive spots are nucleus-limited (Figure 6A), we detected TBPH-positive cytoplasmic spots and trails in MT of *Squid* null mutants (Figure 6B). We confirmed these data in vitro by performing Western blots of nuclear and cytoplasmic fractions in WT versus *Squid* null mutant MT and BCs (Figure 6C,D). In detail, we showed that in *Squid* null mutant the TBPH protein in MT disappears from the nucleus while moving to the cytoplasm, where the TBPH abundance increased compared to the WT NF (+1.74-fold) (Figure 6C’).

We also observed a similar phenomenon in BCs where we found that in *Squid* null mutant TBPH disappear from the NF while its CF amount is quite similar compared to the WT (Figure 6D’). 

Unlike Squid, Hrb87F does not affect TBPH subcellular distribution (Figure S2). To explain this result, we hypothesize the existence of a hierarchical order in omega speckles assembling and we speculated that Squid together with ISWI could be master regulators in the formation of physiologically functional hnRNP-hsrω complexes. In this case we could hypothesize that the loss of Squid protein forces TBPH protein to escape the nucleus as a consequence of incorrect interaction among all omega speckle-associated hnRNPs.

All these data collectively suggest that, in the *Drosophila* cells, the disorganization of omega speckles’ compartments caused by loss of ISWI’s role lead to a redistribution of TBPH protein from the nucleus to the cytoplasm. This could be a very important observation, considering that intracellular deposition of aggregated and ubiquitinated proteins are a prominent cyto-pathological feature of most neurodegenerative disorders frequently correlated with neural cell death. 

To explain all the results presented, we could hypothesize that loss of ISWI’s function may indirectly affect TBPH distribution as a consequence of incorrect interaction among the omega speckle-associated hnRNPs and hsrω arcRNA. Indeed, while Squid and Hrb87F in *ISWI* null mutants are disorganized in their structure, but remain in the nucleus [28], TBPH seems to be more affected and to escape from the nucleus to the cytoplasm.

For instance, our data reinforce the role of the chromatin remodeler ISWI in the modulation of the cellular localization of aggregation-prone proteins and show that the correct nuclear compartmentalization of TBPH hnRNP is dependent on nuclear body maintenance regulated by the chromatin remodeler. Finally, we are convinced that our data are in line with the recent findings showing that TDP-43-dependent reduction of the chromatin remodeler Chd1’s recruitment to chromatin affects the induction of several key stress genes necessary to protect from diseases like ALS and FTD (Frontotemporal Dementia) [37].

## 3. Materials and Methods

### 3.1. Fly Strains and Genetic Interactions

Flies were raised at 25 °C on K12 medium [38]. Unless otherwise stated, strains were obtained from Bloomington Drosophila Stock Center at Indiana University (Dept Biology, Indiana University, 1001 E. Third St., Bloomington, IN, USA). The strain for *hsrω^-^* null mutant (*w^1118^*; *hsrω^66^/TM6B*, *Tb^1^*, Bloo 59617) was obtained from Bloomington Drosophila Stock Center at Indiana University. The *DefHrb87F* and the *sqd^DG09709^* lines are a generous gift from Dr. Subhash Lakhotia. For our analysis, we selected homozygous larvae null mutants from each line (*sqd^DG09709^*/*sqd^DG09709^*, as well as *DefHrb87F/DefHrb87F)* indicated as *Squid^-^* and *Hrb87F^-^.* The *ISWI^2^/T(2;3)* line is a generous gift from Prof. Davide F.V. Corona. We selected the homozygous larvae null mutants *ISWI^2^/ISWI^2^* indicated as *ISWI^-^*.

### 3.2. Antibodies, Plasmid and RNA Probe

Mouse antibodies against the following proteins were used at the indicated dilutions: Hrb87F (P11) [22] dilution 1:50 for immune-fluorescence (IF) and 1:100 for WB for 1 h at room temperature; Squid (1B11) [39] at a dilution of 1:100 for IF and 1:2000 for WB. Rabbit antibody against TBPH protein was used at a dilution of 1:200 for IF and 1:3000 for WB. Rat antibody against Elav protein (DSHB, Developmental Studies Hybridoma Bank University of Iowa, Department of Biology, 028 Biology Building East Iowa City, Iowa 52242-1324) was used at a dilution of 1:250. FITC- and Rhodamine-conjugated anti-mouse and anti-rabbit secondary antibodies (Jackson Immuno Research Laboratories, Inc., 872 West Baltimore Pike, West Grove, PA, USA) were diluted at 1:200 for IF and at 1:5000 for WB. Both were incubated for 1 h at R.T. FITC-conjugated secondary antibody anti-rat 555 (Alexa-Fluor, Thermo Fisher Scientific, 168 Third Avenue, Waltham, MA, USA), was diluted at 1:500 and incubated for 2 h at room temperature. The biotin-labeled anti-sense hsrω-n RNA 280b riboprobe was generated from the *pDRM30* plasmid and used for FRISH and Immuno-FRISH. For IF, all primary antibodies were incubated overnight at 4 °C while secondary antibodies were incubated for 1 h at R.T. For WB analysis, Hrb87F and Squid primary antibodies were incubated for 1 h at 25 °C, while TBPH antibody was incubated overnight at 4 °C. For WB all secondary antibodies were incubated for 1 h at R.T. TBPH antibody [38] is a generous gift from Prof. Frank Hirth.

### 3.3. Immunofluorescence, FRISH, and Immuno-FRISH

Single and double immunofluorescences on polytene chromosomes were conducted as described by [40]. Larval tissues (Malpighian tubules and brains) were dissected from third-instar larvae grown at 25 °C. Fully- or partially-squashed tissue preparations were used for FRISH and Immuno-FRISH assays as previously described [22] with some modifications [28]. For FRISH and Immuno-FRISH, we only use cells from Malpighian tubules as they show a large nuclear size which allow a better understanding of nuclear bodies’ distribution as well as the eventually hsrω-interacting protein subcellular localization, while this is more difficult with cells from other regions, including those from the CNS (central nervous system). Immunostaining on the ventral ganglia dorsal medial clusters of motoneurons in third instar wild-type and *ISWI* null mutant larvae was performed according to standard protocols [41].

### 3.4. Protein Extraction and Western Blotting

Nuclear and cytoplasmic protein extracts from brains and from whole larvae at third instar stage were prepared as described by [33]. Proteins were quantified by Quick Start Bradford 1× dye reagent (BIO-RAD, 1000 Alfren Nobel Drive, Hercules, CA, USA). The blotted membranes were blocked for 1 h at 25 °C, followed by incubation with appropriate primary antibodies. After washing, the membranes were incubated with appropriate secondary antibodies HRP-conjugated: anti-rabbit and anti-mouse (Thermo Scientific, 1201 Wiley Rd, Schaumburg, IL, USA) at 1:2500 and 1:3000 dilutions for 1 h at 25 °C. Antibody binding was detected using SuperSignal West Femto substrate (Pierce, Thermo Fisher Scientific, 168 Third Avenue, Waltham, MA, USA) and chemiluminescent signals were acquired with the ChemiDoc XRS imager (BIO-RAD, 1000 Alfren Nobel Drive, Hercules, CA, USA). We reported the densitometric quantification of protein bands as a ratio with GAPDH and histone H3 to compare proteins levels between WT and *ISWI^-^/Squid^-^* mutants.

### 3.5. Co-Immunoprecipitation

TBPH immunoprecipitation was performed on the nuclear protein extracts prepared as previously described [33] using dynabeads protein A (Novel Life Technologies, Thermo Fisher Scientific, 168 Third Avenue, Waltham, MA, USA) and affinity-purified TBPH antibody. In particular, they were incubated 4 μg of each antibody (TBPH and IgG like negative control. 5 μL of beads were resuspended in 200 μL incubation buffer (10 mM HEPES-KOH pH 8.0, 1 mM EDTA, 10% glycerol, 50 mM NaCl) and washed with IB buffer three times for 10 min in gentle agitation. After the last wash, the beads were incubated in 75 μL IMP buffer for incubation (10 mM HEPES-KOH pH 8.0, 10% glycerol, 100 μg/mL PMSF and Complete 1×) for 2 h at 4 °C in gentle agitation. The beads were washed three times in 200 μL of IB buffer, at the end of last wash, 1/10 of final volume was collected (INPUT). Finally, the beads were incubated with 250 μg of nuclear protein extract and with IMP Buffer (volume 1:1) overnight in gentle agitation. Unbound material was collected; the beads were washed three times for 10 min in IMP buffer for wash (10 mM HEPES-KOH pH 8, 100 mM NaCl, 0.05 tween 20, 10% glycerol, 100 μg/mL PMSF and Complete 1×). In this step, first wash was collected. Finally, the bound material was eluted in 30 μL sodium dodecyl sulfate polyacrylamide gel electrophoresis (SDS-PAGE) sample buffer for Western blotting analysis. For Western blotting, proteins were separated by SDS-PAGE, blotted and challenged with the TBPH antibody, with Hrb87F and Squid antibody. Primary antibody binding was detected using SuperSignal West Femto substrate (Pierce, Thermo Fisher Scientific, 168 Third Avenue Waltham, MA, USA). Chemiluminescent signals were acquired with the ChemiDoc XRS imager (BIO-RAD, 1000 Alfren Nobel Drive, Hercules, CA, USA).

### 3.6. Cross-Linking RNA Immuno-Precipitation (CLIP-RIP)

For CLIP-RIP, 100 μg of nuclear proteins extracts from brains were used. CLIP-RIP was conducted as previously described [42] with some modifications. One-hundred micrograms of nuclear pellet were resuspended in 1× PBS_DEPC_—with 1% formaldehyde and incubated for 15 min under rotation. The crosslinking reaction was quenched by the addition of glycine (pH 7.0) to a final concentration of 0.25 M followed by incubation at room temperature for 5 min. The nuclear pellet was collected by centrifugation at 1100× *g* for 2 min at 4 °C, washed twice with cold 1× PBS_DEPC_. The nuclear pellet was resuspended in 5 mL nuclear extraction buffer (NEB) (15 mM Hepes pH = 7.0, 5 mM MgCl_2_, 0,2 mM EDTA, 0.5 mM EGTA, 10 mM KCl, 350 mM Sucrose (added fresh), 0.1%Tween-20, 1 mM DTT (added fresh) and vortexed for 10 s. NEB was added to make 10 mL volume. The sample was centrifuged at 3220× *g* for 5 min at 4 °C, the supernatant was removed and the cross-linked pellet was resuspended in 2 mL of RIPA buffer (50 mM Tris-HCl pH 7.5, 1% NP40, 0.5% Na-deoxycholate, 0.05% SDS, 1 mM EDTA, 150 mM NaCl) containing protease inhibitors (complete, mini, EDTA-free protease inhibitor cocktail tablet, Roche, F. Hoffmann-La Roche, AG Konzern Hauptsitz, Grenzacherstrasse 124, Basel, Switzerland). The resuspended pellet was sonicated three times for 20 s each with the sample being kept on ice for 2 min between each step of sonication. The insoluble material was removed after centrifugation at 16,000× *g* for 10 min at 4 °C. The RNA was extracted from 25 μL each of the TEL buffer (prepared from 10× TE and 10× LiAc) added to the beads, the unbound or the immunoprecipitated material with 600 μL of TRIzol (Invitrogen, Thermo Fisher Scientific, 168 Third Avenue Waltham, MA, USA); the RNA was precipitated overnight with 0.7 volumes of isopropanol at −20 °C. The pellet was washed in 75% cold ethanol, dissolved in 10 μL RNAse free water and incubated for 15 min at 37 °C.

### 3.7. Real-Time Polymerase Chain Reaction (RT-PCR)

For RT-PCR, the first-strand cDNA was synthesized using MuLV reverse transcriptase (Applied Biosystem Inc., 850 Lincoln Center Drive, Foster City, CA, USA) and 1 μL of random hexamers (Applied Biosystem, Inc., 850 Lincoln Center Drive, Foster City, CA, USA). Four microliters of the reaction mixture was used for PCR amplification using the hsrω-n RNA-specific primer pairs (Invitrogen, Thermo Fisher Scientific 168 Third Avenue, Waltham, MA, USA) that amplify the 280 bp unit of tandem repeats in the hsrω-n transcript (hsrω forward, 5′-CGAAAAGGCTTATCCTCTTGGTAAA-3′, and hsrω reverse, 5′-AAGGATAATGATTAAGGTAATCGGG-3′), the Act5C transcript (ACT5C forward, 5′-CACGGTATCGTGACCAACTG-3′, Act5C reverse, 5′-GCCATCTCCTGCTCAAAGTC-3′), the U4 ncRNA (U4 forward, 5′-GCAGAGGCGATATCGTAACC-3′, U4 reverse, 5′-GCTTCCAAAAATTGCCGTAG-3′) or the Rox1 ncRNA (Rox1 forward, 5′-CCCAGAAGAAACTGCCACTGC-3′, Rox1 reverse, 5′-AATGTCCCTTTTCGAGCG-3′). For PCR amplification of hsrω and Act5C transcripts we used the following program: 94 °C 2 min, 30 cycles (94 °C for 30 s, 50 °C for 30 s, 72 °C for 30 s) and a final extension at 72 °C for 2 min. To amplify the U4 transcript we instead used the following program: 94 °C, 4 min, 30 cycles (at 94 °C for 30 s, at 46 °C for 30 s, at 72 °C for 1 min) and a final extension at 72 °C for 10 min. Finally, for the PCR amplification of the Rox1 transcript, we used: 94 °C 4 min, 30 cycles (at 94 °C for 30 s, at 55 °C for 30 s, at 72 °C for 1 min) and a final extension at 72 °C for 10 min. The PCR products were analyzed by agarose electrophoresis.

### 3.8. Protein Cloning, Expression and Purification

Sixty *D. melanogaster* third-stage whole larvae were dissected to extract the brains from which total RNAs were obtained using TRIZURE^®^ (Bioline, USA Inc., 305 Constitution Drive, Taunton, MA, USA) following the standard procedure. Quality and quantity analyses were performed with BioPhotometer^®^ (Eppendorf North America, 102 Motor Parkwa, Hauppauge, NY, USA), and the purified RNAs were separated through electrophoresis on 1.2% agarose to check integrity. RT-PCR was performed with random hexamers to obtain *D. melanogaster* brain cDNA using a MyTaq™ One-Step RT-PCR Kit (Bioline, USA Inc., 305 Constitution Drive, Taunton, MA, USA). The *TBPH* gene was amplified through PCR from brain cDNA with gene-specific primers Fw: ATG GAT TTC GTT CAA GTG TCG G and Rev: TTA AAG AAA GTT TGA CTT CTC CGC. The PCR product was obtained using following program: 94 °C 2 min, 17 cycles (94 °C for 2 min, 54 °C for 2 min, 72 °C for 2 min) and a final extension at 72 °C for 2 min. PCR product length was checked through electrophoresis on 1.2% agarose gel, then the amplified gene was TA-cloned in the pCR™ 2.1 vector (TA Cloning^®^ Kit with pCR^®^ 2.1 Vector, Invitrogen, Thermo Fisher Scientific 168 Third Avenue, Waltham, MA, USA) and used to transform DH5α *E. coli* cells. The resulting pCR™2.1:TBPH was sequenced to ensure the absent of mutations. Subsequently, the *TBPH* gene was subcloned into the expression vector pGex-6P-3 (GE Healthcare, 41 Farnsworth Street, Boston, MA, USA) using EcoRI and XhoI restriction enzymes (Promega Corporation, 2800 Woods Hollow Road, Madison WI 53711-5399, USA). The resulting recombinant vector was thus used to transform DH5α *E. coli* cells and positive clone was selected through PCR reaction positive for the *TBPH* gene. The positive clone was used for the TBPH protein expression, by induction with IPTG (Isopropil-β-d-1-tiogalattopiranoside) in Super Broth liquid medium (3.5% tryptone, 2.0% yeast extract, 0.5% NaCl, 1N NaOH) (Teknova, 2290 Bert Dr., Hollister, CA 95023). TBPH protein was purified used sepharose matrix by the same kit. The 6× HIS tag was cut using the enzyme Prescission^®^ (GE Healthcare, 41 Farnsworth Street, Boston, MA, USA) and, after, dialyzed using a MILLIPORE^®^ membrane (Millipore, Merck Group Frankfurter Strasse 250, Darmstadt, Germany). Protein samples were checked through Western blot using anti-TBPH.

### 3.9. Gel Mobility Shift Assay

Recombinant full-length TBPH and recombinant full length ISWI were incubated with in vitro transcribed sense 280 bp tandem repeat unit of the *hsr-ω* lncRNA in increasing ratios for TBPH/*hsr-ω* lncRNA of 1:2, 1:50 nM, and a constant ratio for ISWI/*hsr-ω* lncRNA of 1:20. The hsr-ω lncRNA were incubated with the desired proteins for 20 min at 25 °C in RB 2 buffer (20% glycerol, 0.2 mM EDTA, 20 mM Tris-HCl pH 7.5, 1 mM MgCl_2_, 150 mM NaCl, 1 mM DTT and RNAsin). After incubation, the RNA/protein complexes were resolved on 1% agarose gel in 0.5× TBE at 4 °C for 100 min at 70 V. RNA molecules were visualized by ethidium bromide staining.

### 3.10. RNA Samples Preparation

For each *Drosophila* line, 30 brains from third instar larvae were dissected in NaCl 0.7% and collected in TRIsure^®^ (Bioline, USA Inc., 305 Constitution Drive, Taunton, MA, USA). After TRIsure^®^ RNA extraction, samples were treated with *RNase-free* DNase I (Thermo Scientific, 1201 Wiley Rd, Schaumburg, IL, USA) using 1 U of DNase for 1 μg of RNA as determined by spectrophotometry at 260 nm. To stop the reaction and purify the RNA, phenol-chloroform extraction was conducted with a standard protocol. Samples were analyzed with an (D30 Biophotometer Eppendorf North America, 102 Motor Parkwa, Hauppauge, NY, USA) and all showed perfect A260/A230 and A260/A280 ratios.

### 3.11. Images Acquisition and Quantification

Images were acquired under a confocal laser-scanning microscope Zeiss, LSM 510 META (Zeiss, Via Varesina 162, 20156 Milano, MI, Italia) by using 63× oil immersion and 40× lenses. To quantify and compare fluorescence across images and bands, raw files were analyzed using Fiji software [43] as previously described [44]. An Unpaired Student’s *t*-test was performed to assay statistical significance. In all images, a single asterisk denotes a *p*-value between 0.01 and 0.05. Two asterisks denote a *p*-value less than 0.05. Where asterisks are not shown, results of statistical tests were greater than 0.05.

## 4. Conclusions

In this study, we revealed that TBPH, the *Drosophila* homolog of mammalian TDP-43, is a component of the omega speckles’ nuclear body compartment. TBPH interacts with the arcRNA hsrω and omega speckle-associated hnRNPs in vivo and in vitro. We also showed that the loss of chromatin remodeler ISWI’s function drastically changes TBPH sub-cellular compartmentalization, driving the aberrant TBPH cytoplasmic redistribution even in absence of TBPH protein mutation. 

In several neurodegenerative diseases, the pathology and eventual death of specific neuronal populations is associated with the accumulation of distinctly abnormal polypeptides [45]. As the altered sub-cellular TBPH distribution could globally affect RNA processing, RBPs aggregation could represent the link with problems in RNA metabolism. Indeed, the disruption of their proper localization may result in the loss of their normal function, as well as alterations in RNA transport and splicing, which are crucial in neuronal health. Considering that the function of RBPs is finely regulated and generally depends on their subcellular localization, the fact that ISWI could regulate TBPH subcellular localization opens a new scenario in which the loss of the chromatin remodelers’ function may be involved in the onset of neurological disorders even in the absence of RBP mutations. Moreover, as TDP-43 could affect at a chromatin level the correct expression of stress genes involved in several neurological diseases, chromatin remodelers, like ISWI, may play critical key roles in these pathways. 

Given this, further analysis will be necessary for a precise characterization of the molecular nature of the interaction existing between omega speckles, TBPH, and chromatin remodelers, like ISWI. Indeed, RNA processing and post-transcriptional regulation mediated by chromatin remodelers constitutes a common theme for several neurodegenerative disorders that are characterized by abnormal protein inclusions. Gaining a better knowledge of the molecular events underlying the deregulation of hnRNPs activity between nucleus and cytoplasmic compartments catalyzed by chromatin remodelers is a mandatory step to develop and improve new therapeutic targets and strategies against several devastating neurodegenerative diseases.

## Supplementary Materials

Supplementary materials can be found at http://www.mdpi.com/1422-0067/19/4/1082/s1. Figure S1. Anti-hnRNPs antibodies specificity control. (A–F’’) The anti-TBPH, anti-Squid (1B11) and anti-Hrb87F (P11) recognize specifically each hnRNP on polytene chromosomes (B–B’–B’’), respect to each null mutant (E–E’–E’’). Figure S2. Loss of Hrb87F does not cause change in TBPH localization. (A,B) Loss of Hrb87F does not change TBPH protein (red) distribution between nucleus and cytoplasm in MT of *Hrb87F^-^* null mutants (B,C) compared to WT (A). Indeed, TBPH is co-localized with hsrω (green) and it is predominantly distributed in the nucleus. (C,D) Western blots confirming the data for MT cells (C) as well as BCs (D). The intensity of Western blot signals was quantified using ImageJ. The experiment was performed considering five biological replicates. The error bars show the standard deviation. Unpaired Student’s *t*-test was performed to assay statistical significance; * 0.01 ≤ *p*-value ≤ 0.05; ** *p*-value < 0.01.
