# Supplementary figures and images for: Loss of ISWI Function in Drosophila Nuclear Bodies Drives Cytoplasmic Redistribution of Drosophila TDP-43
Source: Int J Mol Sci. 2018 Apr 4;19(4):1082. doi: 10.3390/ijms19041082 (PMC5979594; doi:10.3390/ijms19041082)

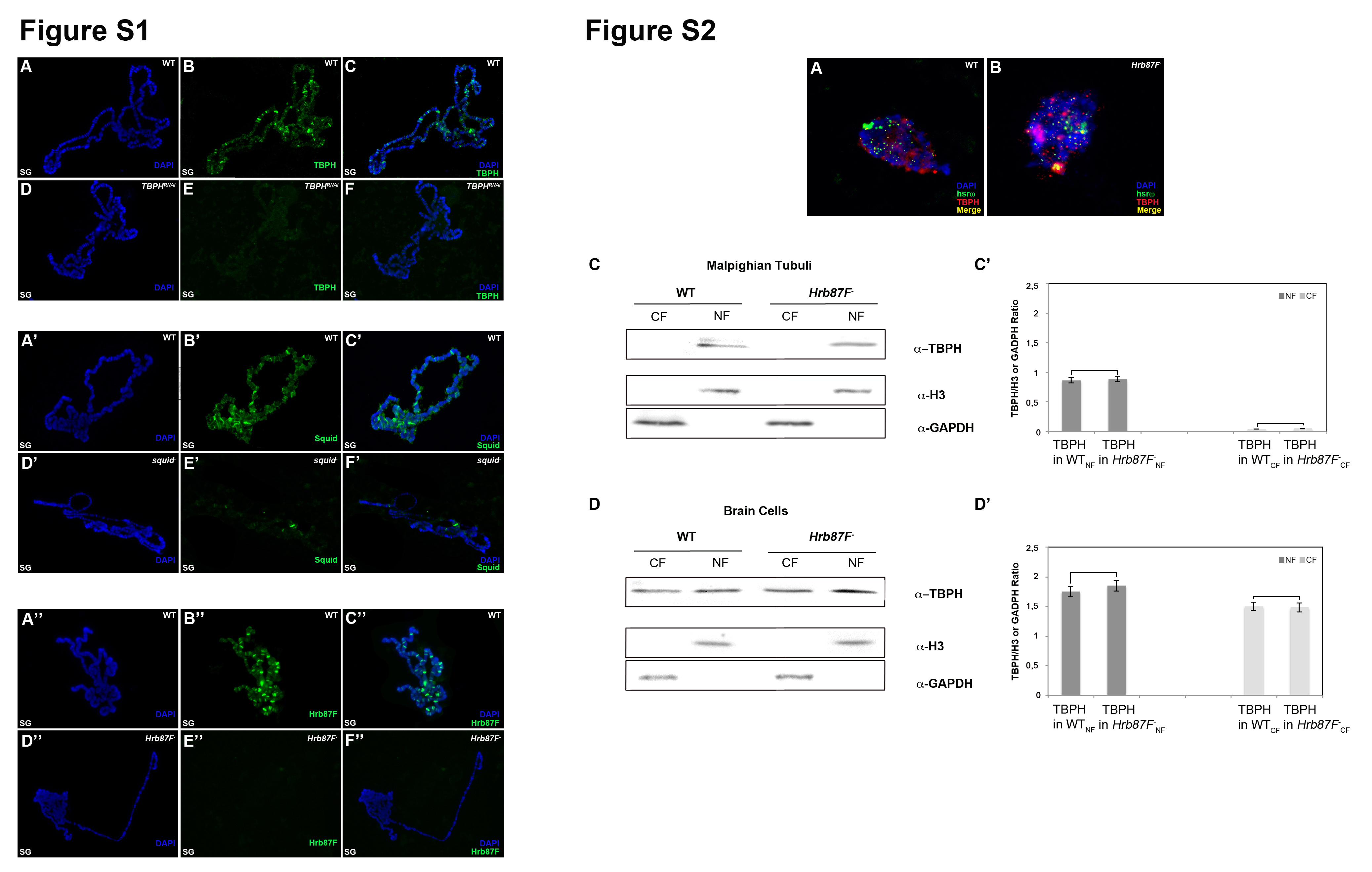

Supplement: Supplementary file 1 [file ijms-19-01082-s001.jpg]
